# Benner's Model Guiding Skill and Knowledge Acquisition of Advanced Practice Nurses

**DOI:** 10.1111/nup.70078

**Published:** 2026-03-30

**Authors:** Maya Zumstein‐Shaha

**Affiliations:** ^1^ Bern University of Applied Sciences Health, Master of Science in Nursing Bern Bern Switzerland; ^2^ Department of Nursing, Faculty of Health University of Witten/Herdecke Witten Nordrhein‐Westfalen Germany; ^3^ Faculty of Health & Social Sciences Bournemouth University Bournemouth Dorset UK; ^4^ Anschutz Medical Center, College of Nursing University of Colorado Denver Colorado USA

**Keywords:** Advanced Practice Nurses, Benner's model, From Novice to Expert, Nursing expertise, Philosophy of science

## Abstract

In 2016, Bowen and Prentice published a dialogue on Benner's model ‘From Novice to Expert’. Their main concern was to explore the development of expertise in nursing and the evolution of this expertise when domains of practice change. In this contribution, this dialogue by Bowen and Prentice (2016) is the starting point to consider Advanced Practice Nurses (APN). The argument will propose that Benner's model can help perceive APN skill (and knowledge) acquisition. For this purpose, Benner's original works, the philosophical bases on knowing that and knowing how, as well as the concept of APN, which was developed by Ann Hamric (2014; Tracy et al. 2023), are consulted. Eventually, it will be demonstrated that Benner's model can be employed in conjunction with APN and can provide valuable insight into skill and knowledge acquisition and career development. Therefore, APN can reinvigorate Benner's model and render it again very valid for today. This contribution will conclude with suggestions for supporting APNs' skill and knowledge development and indications for the evolvement of Benner's model.

## 1 Introduction

Demographic changes in populations around the globe demand adaptations in healthcare, healthcare professionals (HCP), their respective education and career pathways. Among these changes, the role of the Advanced Practice Nurses (APN) is becoming increasingly important (Altermatt‐Von Arb et al. 2023; Pol‐Castañeda et al. 2022; Schlunegger et al. 2023). The term APN encompasses roles such as ‘Nurse Practitioners’, ‘Clinical Nurse Specialists’, ‘Certified Nurse Midwives’ and ‘Certified Registered Nurse Anaesthetists’, depending on the respective country (APRN Consensus Work Group and National Council of State Boards of Nursing APRN Advisory Committee 2008; Mahrer‐Imhof et al. 2024). For nurses to become APNs, they have to obtain at least a Master of Science in Nursing (MSN) degree and subsequently work with patients and their families in clinical practice. As part of their scope of practice, APNs demonstrate advanced skills in nursing, clinical assessment and reasoning, coaching and consulting of various HCP. They provide leadership, collaborate with the interdisciplinary team, engage in ethical decision making, and base their practice on the latest evidence (Hamric et al. 2014; Tracy et al. 2023). APNs are trained to provide a combination of advanced nursing and medical skills for person‐centred care to specific patient groups. These patients require a new model of care as they currently encounter gaps in needs‐based care as well as under‐, over‐ or mistreatment (Eissler and Zumstein‐Shaha 2022; Hamric et al. 2014; Tracy et al. 2023; Zumstein‐Shaha et al. 2022). Depending on existing and respective national and local regulations, APNs can work independently within their scope of practice but often interact and collaborate closely with physicians (Eissler and Zumstein‐Shaha 2022; Zumstein‐Shaha et al. 2022; Zumstein‐Shaha et al. 2024). For the role of APN, these professionals require a broad range of knowledge from nursing, medicine, and other related scientific disciplines. In this contribution, the argument will be made that Benner's model ‘From Novice to Expert’ can be used to explore skill and knowledge acquisition in APN. This argument is interesting to explore, considering APNs started out as novice nurses at some point. For this purpose, the contribution by Bowen and Prentice (2016) will serve as a starting point, followed by a brief presentation on APN and a snapshot of Benner's model of skill acquisition in nursing (2001, 2004, 2019; Benner et al. 2009). In the discussion, the argument will be explored as well as the importance of Benner's model for explanatory purposes. The conclusion will provide indications for APNs' skill development and also address the role of Benner's model.

## 2 Near Extinction of Benner's Expert Nurses

The title of Bowen and Prentice's (2016) dialogue is provoking. The authors suggest that the ideas of Benner's model ‘From Novice to Expert’ do no longer hold as the introduction of digital transformation renders nursing and related knowledge processes almost expendable. To illustrate this point, the authors draw on their own personal experience in Labour and Delivery at a large Canadian hospital. The collaboration with experienced nurses makes the authors realise that these nurses often stated that they ‘just knew what to do in a situation’ (Bowen and Prentice 2016, p. 145) in lieu of a more comprehensive explanation. In consideration of Benner's model, Bowen and Prentice (2016) conclude that these experienced nurses were representatives of the ‘expert nurse’. With the advent of digital transformation in the form of new technology and digitalised patient records, changes occurred in daily nursing care. Guideline‐driven and standardised practice grew more important along with measurable outcomes, which became the new currency by which healthcare and professional performance were assessed. As a result, these expert nurses needed new knowledge and skills. Therefore, they again felt like novices in their field. The digital transformation also necessitated guidelines, “standing orders, medical directives, and algorithms of care” (Bowen and Prentice 2016, p. 146). Experiential knowledge acquired through daily work with patients became more and more restricted, limiting the possibility of honing one's knowledge and skills in practice and developing clinical judgement. The dynamic nature of change in healthcare was perceived to threaten nursing care, which requires daily work and engagement with patients and their families. Thus, nurses can develop an intuitive grasp of any situation, which can be viewed as one of the main perks and attractions of nursing. As a result, digital healthcare transformation was perceived to limit nursing care substantially, thereby rendering it less attractive for future nurses.

According to Benner's model (2001, 2004, 2019; Benner et al. 2009), expert nurses can quickly, almost automatically grasp a situation comprehensively. These nurses draw on their extensive knowledge, including existing guidelines, standards, directives and their grown clinical experience and judgement. As a result, expert nurses know what to do in most situations they encounter. They address patient issues in a timely, individualised, but adequate way. Thus, these expert nurses obtain improved patient outcomes.

Ultimately, expert nurses are less and less likely to emerge. A major attraction of the nursing profession will be lost, which will impact recruitment and retention. Ultimately, patient care and outcomes will suffer. Bowen and Prentice (2016) advocate for a re‐evaluation of current practices to ensure that the development of nursing expertise is supported and valued within modern healthcare settings. In this contribution, this less‐than‐encouraging view constitutes the starting point to explore the situation of APN. It is argued that the knowledge and skill acquisition of professionals in the roles of APN also needs to be explored in order to promote their career pathways and professionalisation.

## 3 Advanced Practice Nurses

This role, for which an international guideline exists, is becoming increasingly important for healthcare globally (Altermatt‐Von Arb et al. 2023; International Council of Nurses ICN 2020; Pol‐Castañeda et al. 2022). To become an APN, nurses have to complete at least a Master of Science in Nursing (MSN) degree, obtaining a large(r) knowledge base and new skills. Such professionals are expected to clinically work with patients and their families. In particular, persons with chronic diseases and multimorbidity currently experience gaps in needs‐based care as well as under‐, over‐ or mistreatment (Eissler and Zumstein‐Shaha 2022; ICN 2020; Tracy et al. 2023; Zumstein‐Shaha et al. 2022; Zumstein‐Shaha et al. 2024). APNs work independently and are trained to provide a combination of advanced nursing and medical skills for person‐centred care to specific patient groups. They demonstrate advanced skills in coaching and consulting of nurses and HCP, leadership, collaboration, ethical decision making, and evidence‐based practice (Hako et al. 2023; Tracy et al. 2023). APNs employ their high level of knowledge and skills to creatively and adequately adapt evidence‐based care to the specific situation of each patient, their relatives and specific patient groups (Eissler and Zumstein‐Shaha 2022; Hako et al. 2023; Zumstein‐Shaha et al. 2022; Zumstein‐Shaha et al. 2024).

Although being a nurse is the foundation for an APN, the training, scope of practice and level of independent work differ substantially. Moving into the APN role—after having worked as a nurse—can signify a ‘transition shock’ (Fitzpatrick and Gripshover 2016). New APNs can experience uncertainty, ambiguity, anxiety, and general instability until having completely adapted to the new role (Faraz 2016; Fitzpatrick and Gripshover 2016).

Due to changes in healthcare, the number of APNs is expected to increase (Auffermann et al. 2021; Finneran and Kreye 2021). The APN role is perceived to differ substantially from the role of the registered nurse. Therefore, new APN experience role transition (Auffermann et al. 2021; Finneran and Kreye 2021). This transition is defined as: “a process consisting of multiple mixed emotions that occurs over time, and is a period of great personal development and learning as the NP takes on new autonomy and responsibility for patients.” (Barnes 2015: 142). When unsuccessful, novice APNs are more likely to be dissatisfied and ultimately leave their jobs (Finneran and Kreye 2021). As part of their new role, APNs try to unite the experience as a registered nurse with the new position, which differs from a physician's role. However, clearly demarcating between the APN's and physicians' roles can be challenging (Cheng et al. 2022). This is mainly due to the fact that APNs have developed a combination of advanced nursing and medical skills, such as conducting a thorough health assessment (Zumstein‐Shaha et al. 2022; Zumstein‐Shaha et al. 2024). In addition, in many countries, the scope of practice of APN is still in need of refinement, which can also prevent clear demarcation between APN and physicians (Zumstein‐Shaha et al. 2022; Zumstein‐Shaha et al. 2024).

APN work includes making or adjusting prescriptions, which can change current treatments. In the new role, APNs need to acquire experience and build relationships (Cheng et al. 2022). Therefore, the APN starts as a novice in the new role, which can be destabilising and stressful. Attached uncertainties can provoke self‐doubt (Barnes 2015; Cusson and Strange 2008). APN may “feel like an impostor” (Barnes 2015: 140). Decision making as an APN raises anxieties and uncertainties due to the responsibility for the consequences (Cusson and Strange 2008). APNs realize that they are facing many expectations from other HCPs, patients and relatives (Cheng et al. 2022). Critique and support from other HCP can contribute or hinder the successful transition from registered nurse to APN (Barnes 2015; Cusson and Strange 2008). The majority of the transition burden appears to fall into the first year as a novice APN (Cusson and Strange 2008).

To date, only a few orientation or transition programs or fellowships have been established for novice APNs. Some of these programs refer to Benner's model (2001, 2004, 2019; Benner et al. 2009). Generally, novice APNs seem content in their jobs. However, intention to leave remains high within the first 2 years of employment (Auffermann et al. 2021; Gourley et al. 2023; Hande and Jackson 2025). To adapt to the new situation and the role demands, APNs use established coping strategies. Where necessary, they acquire new coping strategies (Cheng et al. 2022). Mentoring and orientation programs can contribute to faster adaptation of novice APNs to their work (Cusson and Strange 2008). Such programs also provide APNs with mentoring skills. As a result, mentoring programs enable APNs to mentor junior colleagues in the same role or other HCPs (Dunlap and Fitzpatrick 2023; Ellerbe and Regen 2012).

After adjusting to the new role, APNs often become key players within an institution and in the healthcare system. Recognition of this contribution is necessary as well as continued education. Specifically developed career ladder programs that are also based on Benner's model (2001, 2004, 2019; Benner et al. 2009) are well established for registered nurses (Auffermann et al. 2021; Barnes 2015; Cusson and Strange 2008; Faraz 2016; Fitzpatrick and Gripshover 2016; Finneran and Kreye 2021). However, such programs have yet to be systematically implemented for APN (Jnah and Robinson 2015; Kauffman et al. 2022). When linked to specific criteria such as levels of education and training as well as salary, career ladder programs ultimately contribute to professional growth and development (Jnah and Robinson 2015; Kauffman et al. 2022).

In summary, the transition from registered nurse to APN has been studied in more detail. However, gaps remain concerning the acquisition of APN skills and knowledge from the moment of entry into the role throughout the career. With the growing number of APNs in countries such as the United States (USA), and the increasing interest in the APN role of countries such as Switzerland, it is interesting to consider APN skill and knowledge acquisition throughout their career. For this purpose, Benner's model will be presented below and discussed in relation to APN.

## 4 Benner's ‘From Novice to Expert’ and APN

This model builds on the one by Stuart E. and Hubert L. Dreyfus, which is in turn based on studies with chess players, air force pilots, army tank drivers, and commanders (Benner 2004; Benner et al. 2009). For her own model, Benner (2004, 2019) conducted three studies with nurses across a time span of 20 years. Data revealed that nurses' skill and knowledge acquisition follow the posits of the Dreyfus model. Similarly, Benner's model encompasses five levels of skill acquisition. Associated knowledge acquisition is implied. However, Table 1 below proposes a combination of skill and knowledge acquisition.

**Table 1:** Skill and knowledge acquisition in benner's model.

Level	Novice	Advanced beginner	Competent	Proficient	Expert
Skill acquisition	Nursing education, training Exposure to clinical practice under direct supervision	Nursing education, training Practicing with direct and indirect supervision	Textbook learning Preparing, organizing and managing work under indirect supervision or independently	Managing routine situations well Recognizing routine situations	Full and immediate grasp of the situation Adjusting to the situation's demands Adapting individual patient care
Knowledge acquisition	Learning context‐free rules to guide action	Learning from guidelines, standards, directives and general principles	Learning from decision‐making games or simulations Adapting to changes Having a heightened awareness of responsibility	Learning from accumulated clinical experiences and through exemplars Synthesizing patient meanings, theoretical knowledge, and clinical experience Grasping the situation as a whole	Learning from interprofessional collaboration Fully grasping the situation as a whole Employing intuition through embodied know‐how paired with local, specific knowledge, technical and scientific knowledge
Timeline	Years 1 and 2 of the nursing training	Year 3 of the nursing training incl. year 1 as a registered nurse (RN)	Years 2 and 3 as an RN	Years 3 and 4 as an RN	Years 5+ as an RN

*Note:* Direct supervision signifies that the student or new nurse works with an experienced nurse, or that the experienced nurse at hand. Indirect supervision means that the experienced nurse is next door and can be fetched when necessary. For an overview of Benner's ‘From Novice to Expert’, see Benner (2019).

Nursing school provides the foundation of skills and knowledge for new nurses. Upon entering into the role, RN need to continue to build knowledge and skills on topics they encounter. Also, technology advances, new evidence emerges, and experience grows through clinical practice. According to Benner (2001, 2004, 2019; Benner et al. 2009), nurses are fully aware of these demands and comply by engaging in educational activities throughout their careers (see Table 1). Nurses also evolve professionally by engaging in critical reflection. Thus, they are able to introduce corrective measures where necessary to improve risk and danger recognition as well as preventing perpetuation of mistakes. Ultimately, nurses may be able to immediately grasp a situation intuitively, while providing individualised patient care (Benner 2001, 2004, 2019; Benner et al. 2009). ‘Intuition’ is described as “we can know more than we can tell” (Polanyi and Sen 2009, p. 4). A person may fully grasp any given situation or act in some way or another appropriately in a very rapid if somewhat automatic manner (Polanyi and Sen 2009). When challenged, persons find giving a clear explanation challenging (Gascoigne and Thornton 2014).

In Benner's model (2001, 2004, 2019; Benner et al. 2009), critical reflection is essential to move from one level to the next. Growing experience in clinical settings is not considered sufficient for this development. Therefore, building critical reflection and integrating it into clinical practice are key for nurses to build skills and knowledge. According to Benner's model (2001, 2004, 2019; Benner et al. 2009), novice nurses do not possess intuition. However, expert nurses are defined by intuition as they are described to being able to fully grasp a situation and act in adequate ways, even without being able to immediately fully explain the underlying rationale (Benner 2001, 2004, 2019; Benner et al. 2009). Consider the following example: (Table 2).

**Table 2:** Example on intuition in nursing.

Sandra is 59 years old and is hospitalized in a palliative care unit. She is being cared for by Ellie, an experienced nurse who can be considered an expert according to Benner (2001; 2004, 2019; Benner, et al. 2009). At start of shift, Ellie is told that the team believes Sandra to be at the end of her life but death is not yet imminent. After having met Sandra, Ellie is convinced that death is imminent. Ellie calls Sandra's family, and they all arrive at her beside. Sandra passes away, surrounded by her family. Only a few hours later, the expert nurse is able to reflect on her reaction and to talk about this experience with other nurses and healthcare professionals.

Bowen and Prentice (2016) suggest that Benner's expert nurses rely almost entirely on experiential learning. According to Benner (2001, 2004, 2019; Benner et al. 2009) nurses need a combination of technological, evidence‐based and experiential knowledge throughout their career. These types of knowing can be related to the philosophical types of knowing called ‘knowing that’ or empirical knowledge, ‘knowing how’ or practice knowledge, and ‘knowing how it is’ or phenomenal knowing (Baumann 2015). Empirical knowledge encompasses evidence‐based knowledge, such as guidelines. This type of knowledge has evolved from research and can be tested in exams. Practice knowledge seems to be reflected in the technological knowledge, such as employing certain devices or giving a bed bath. Experiential knowledge proposed by Benner (2001, 2004, 2019; Benner et al. 2009) can be associated with phenomenal knowledge. This last type of knowledge appears to be linked predominantly with the nurses' own experience of practice, such as having cared for several persons who are at the end of their lives. Although many other nurses may have this same exact experience, for each of them the knowing how it is can differ (Kisanuki et al. 2024). According to Benner (2001, 2004, 2019; Benner et al. 2009), a combination of these three types of knowledge is necessary to provide adequate and tailored patient care. It can be assumed that the better this combination is attained, the better patient care is provided.

Expert nurses seem to possess another type of knowledge, namely intuition, which can also be called ‘tacit knowledge’ (Polanyi and Sen 2009). For Polanyi and Sen (2009), intuition is a new type of knowledge that requires several years of experience in a particular field. Intuition cannot be subsumed by or obtained through other types of knowledge, such as ‘knowing that’, ‘knowing how’ or ‘knowing how to’. Reproducing intuition, from this perspective, is challenging and questionable. In contrast, Gascoigne and Thornton's (2014) intuition can be considered as a form of ‘knowing that’. It is argued that a certain amount of empirical knowledge is essential in order for a person to grasping something rapidly. In other words, intuition is a kind of well‐digested and very well‐established ‘knowing that’ (Gascoigne and Thornton 2014). According to Benner (2001, 2004, 2019; Benner et al. 2009), intuition not only includes knowledge but also decision making and undertaking specific actions, as is demonstrated in the example above. Therefore, a certain amount of ‘knowing how’ seems present. At the expert nurse level, knowledge acquisition includes “(1) developing the skill of involvement, (2) managing technology and preventing unnecessary technological intrusion, and (3) working with and through others.” (Benner et al. 2009, p. 160). Considering the above example, the expert nurse also evidences ‘knowing how something is’ as she calls the family to be present. Drawing on the above example, intuition appears to include a combination of ‘knowing that’, ‘knowing how’ with ‘knowing how something is’. Although a certain amount of ‘knowing that’ is present, expert nurses are challenged to provide rationales and explanations (Benner 2001, 2004, 2019; Benner et al. 2009). Nurses demonstrating intuition not only automatically recognise the situation at hand, but they also know how to act timely, adequately as well as tailored to the patient's preferences (see Table 2). These actions are generally beneficial for the patients (Benner 2019; Chung and Jung 2024) (Table 3).

**Table 3:** ‘Knowing That’, ‘Knowing How’, ‘Knowing How It Is’, and Intuition.

Type of knowing	Propositional knowing	Practice knowing	Phenomenal knowing	Intuition
Alternate title	Knowing that	Knowing how	Knowing how it is	Tacit knowing
Description	Evidence‐ and data‐based knowledge that is obtained through learning from textbooks, etc.	Skill of doing something, repetitively executing a task or an action	Bodily experience of a certain task, action, something	Knowing more than one can tell
Example	Being knowledgeable about the pathology, diagnosis, treatment, and nursing follow‐up of heart failure, and the latest evidence‐based guidelines to treat this condition,	Being able to conduct a clinical heart and lung assessment including a nursing assessment	Having cared for various heart failure patients in the past, and thus being more attuned to the associated changes in health	Immediately grasping the deterioration in a heart failure patient, and take adequate action such as improve positioning, check medication, maybe even administer medication, call for a physician where necessary

*Note:* Table based on Baumann (2015) and Polanyi and Sen (2009).

## 5 Benner's Model in Relation to APN

To become an APN, nurses have to obtain at least an MSN. It is stated that moving from the RN into the APN role does not happen automatically after the completion of the academic degree. Rather, APNs undergo transitioning periods where they learn about the work in this new role, learn the extent of the scope of practice, and adjust to the level of independence associated with this role (Barnes 2015; Cheng et al. 2022; Cusson and Strange 2008; Finneran and Kreye 2021; Fitzpatrick and Gripshover 2016). Drawing on Benner's model, experiences such as these seem similar to the ones of novice nurses. The MSN is for APN what nursing school is for nurses. Similarly, the first year after the MSN can be compared with the nurses' first year after nursing school. During this period, novice nurses learn ‘the ropes’ and face the associated level of responsibility. In contrast, the level of responsibility of APN is broader with more consequences (Hamric et al. 2014; Tracy et al. 2023). It is expected that APN develop in their roles, and build a specific knowledge concerning specific patient groups (Eissler and Zumstein‐Shaha 2022; Zumstein‐Shaha et al. 2022; Zumstein‐Shaha et al. 2024). Similarly, nurses moving from novice through the stages to expert build their skills and knowledge. Nurses often continue in one specific setting with specific patient groups. In that, they may develop a speciality area such as working in cardiology or palliative care. Thus, they are able to strengthen and extend their ‘knowing that’ about the specific patient group, ‘knowing how’ when performing tasks and actions with the specific patient group in the respective setting, and to obtain ‘knowing how it is’ during this time. Similarly to nurses, APNs continue working in one setting (Eissler and Zumstein‐Shaha 2022; Zumstein‐Shaha et al. 2022; Zumstein‐Shaha et al. 2024). In their new role, APN may be supervised by fellow APNs or physicians. This supervision seems heterogeneous. In the USA, there are expectations that a novice APN is able to perform their work when entering the role. Other countries, such as Switzerland, require a certain supervisory period dependent on the respective physicians or fellow APN (Eissler and Zumstein‐Shaha 2022; Zumstein‐Shaha et al. 2022; Zumstein‐Shaha et al. 2024). In that, APNs differ slightly from nurses, and national regulations may apply. At some point, even under supervision, APNs conduct a thorough health exam, decide on a diagnosis, propose medical and nursing treatments for the specific patient, and conduct follow‐ups. This kind of autonomy is different from a nurse who rather performs tasks and actions as delegated by the physicians, more advanced nurses, APN, or even other HCP. Another difference between nurses and APN is the level of responsibility. As this is difficult to grasp theoretically, APN require time to understand the responsibility and take it on (Barnes 2015; Cheng et al. 2022; Cusson and Strange 2008; Finneran and Kreye 2021; Fitzpatrick and Gripshover 2016). Therefore, even expert nurses turn novice APN upon completing an MSN (Barnes 2015; Cheng et al. 2022; Cusson and Strange 2008; Finneran and Kreye 2021; Fitzpatrick and Gripshover 2016). Similar to nurses, it is expected that APN continue acquiring knowledge and skills throughout their careers. Therefore, adequate career plans and support are proposed, which draw on Benner's model (2001, 2004, 2019; Benner et al. 2009). In contrast to nurses, mentoring is recommended for novice APN as an important tool to grow professionally (Auffermann et al. 2021; Dunlap and Fitzpatrick 2023; Faraz 2016; Finneran and Kreye 2021; Fitzpatrick and Gripshover 2016). There are indications that APN need to demonstrate continued education throughout their career, depending on national regulations (Hamric et al. 2014; Tracy et al. 2023). APNs are also expected to engage in publication efforts, and to participate in research conferences. In this particular aspect, APN differ also from nurses. Transitioning into the APN role requires careful planning, role delineation, and mentoring to be successful (Barnes 2015; Dunbar et al. 2019; Fitzpatrick and Gripshover 2016; Gourley et al. 2023; Jnah and Robinson 2015; Kauffman et al. 2022). It seems that Benner's model (2001, 2004, 2019; Benner et al. 2009) also has relevance for APN. Particularly, providing a career pathway for skill and knowledge acquisition throughout the APN career can be established by drawing on this model.

## 6 Conclusion

Bowen and Prentice (2016) focused on the transition from expert nurses to novice nurses as a result of changes in the immediate clinical environment. Due to the growing importance of digital transformation, Bowen and Prentice (2016) were concerned that the essence of nursing was endangered. As a result, they feared for the role of the expert nurse.

However, when nurses become APNs, there is a transition to novice. In the role of APN, these persons are also required to build skills and knowledge throughout their career in order to provide the high level of care associated with this role (Hande and Jackson 2025; Pol‐Castañeda et al. 2022). Current programs for APN career development also draw on Benner's model (Jnah and Robinson 2015; Kauffman et al. 2022). It can be concluded that the expert level constitutes an attractive goal for APN students and graduates. However, further exploration of the pathway of skill and knowledge acquisition of APN is warranted to improve current career pathways and to promote implementation of such programs (Barnes 2015; Dunbar et al. 2019; Fitzpatrick and Gripshover 2016; Gourley et al. 2023; Jnah and Robinson 2015; Kauffman et al. 2022). In addition, further exploration of Benner's model is warranted to promote adaptation to the APN role.

## Funding

The authors received no specific funding for this work.

## Ethics

No ethics statement was necessary for this manuscript as it is entirely based on literature.

## Conflicts of Interest

The author declares no conflicts of interest.

## Data Availability

This manuscript is based on the literature listed in the bibliography.
